# The Relationship of IL-8 and IL-10 Myokines and Performance in Male Marathon Runners Presenting Exercise-Induced Bronchoconstriction

**DOI:** 10.3390/ijerph17082622

**Published:** 2020-04-11

**Authors:** Juliana de Melo Batista dos Santos, André Luis Lacerda Bachi, Luiz Antonio Luna Junior, Roberta Foster, Ana Paula Renno Sierra, Marino Benetti, José Roberto Araújo, Nabil Ghorayeb, Maria Augusta Peduti Dal’Molim Kiss, Rodolfo P. Vieira, Dominique M. A. Bullens, Mauro Vaisberg

**Affiliations:** 1 Department of Otorhinolaryngology, ENT Lab, Federal University of São Paulo (UNIFESP), São Paulo 04025-002, Brazil; allbachi77@gmail.com (A.L.L.B.); luna.junior1980@gmail.com (L.A.L.J.); robertafoster@ig.com.br (R.F.); vaisberg.mauro@gmail.com (M.V.); 2 Post-Graduation Program in Health Sciences, Santo Amaro University (UNISA), São Paulo 04829-300, Brazil; 3 Method Faculty of Sao Paulo (FAMESP), São Paulo 04046-200, Brazil; 4 School of Physical Education and Sport, University of São Paulo (USP), São Paulo 05508-030, Brazil; anasierra@usp.br (A.P.R.S.); marinoben@hotmail.com (M.B.); mapedamkiss@gmail.com (M.A.P.D.K.); 5 Department of Morphology and Genetics, Federal University of Sao Paulo (UNIFESP), São Paulo 04023-900, Brazil; roberto.unifesp@gmail.com; 6 Sports Cardiology Department, Dante Pazzanese Institute of Cardiology, São Paulo 04012-909, Brazil; nghorayeb@cardioesporte.com.br; 7 Post-Graduation Program in Sciences of Human Movement and Rehabilitation, Federal University of São Paulo (UNIFESP), Santos 11060-001, Brazil; rodrelena@yahoo.com.br; 8 Post-Graduation Program in Bioengineering and Biomedical Engineering, Universidade Brasil, São Paulo 08230-030, Brazil; 9 School of Medicine, Anhembi Morumbi University, São José dos Campos 04705-000, Brazil; 10 Brazilian Institute of Teaching and Research in Pulmonary and Exercise Immunology (IBEPIPE), São José dos Campos 12245-520, Brazil; 11 Clinical Division of Pediatrics, UZ Leuven, 3000 Leuven, Belgium; dominique.bullens@kuleuven.be; 12 Allergy and Clinical Immunology Research Group, Department of Microbiology, Immunology and Transplantation, KU Leuven, 3000 Leuven, Belgium

**Keywords:** pulmonary function, endurance exercise, EIB, cytokines, FEV1, FVC, aerobic capacity

## Abstract

At present, it is unclear which exercise-induced factors, such as myokines, could diminish the negative impact of the reduction in pulmonary function imposed by the exercise in question. In this study, we aim to evaluate the prevalence of exercise-induced bronchoconstriction (EIB) and also to investigate the effect of myokines in the performance of marathon runners presenting EIB or not. Thirty-eight male recreational marathon runners (age 38.8 [33–44], height 175.7 [172.0–180.3]; weight 74.7 [69.3–81.6]) participated in this study, and through spirometry tests, a prevalence of 23.6% of EIB was found, which is in agreement with the literature. The volunteers who tested positive to EIB (EIB+) presented lower maximum aerobic capacity compared to those who tested negative (EIB−) (EIB+ 44.02 [39.56–47.02] and EIB− 47.62 [44.11–51.18] *p* = 0.03). The comparison of plasma levels of IL-1β (EIB+ *p* = 0.296, EIB− *p* = 0.176, EIB+ vs. EIB− baseline *p* = 0.190 immediately after *p* = 0.106), IL-4 (undetectable), IL-6 (EIB+ *p* = 0.003, EIB− *p* ≤ 0.001, EIB+ vs. EIB− baseline *p* = 0.301 immediately after *p* = 0.614), IL-8 (EIB+ *p* = 0.003, EIB− *p* ≤ 0.001, EIB+ vs. EIB− baseline *p* = 0.110 immediately after *p* = 0.453), IL-10 (EIB+ *p* = 0.003, EIB− *p* ≤ 0.001, EIB+ vs. EIB− baseline *p* = 0.424 immediately after *p* = 0.876) and TNF-α (EIB+ *p* = 0.003, EIB− *p* ≤ 0.001, EIB+ vs. EIB− baseline *p* = 0.141 immediately after *p* = 0.898) were similar in both groups 24 h before and immediately after the marathon. However, negative correlations were found between the marathon finishing time and the levels of IL-8 (r = −0.81, *p* = 0.022), and IL-10 (r = −0.97, *p* ≤ 0.001) immediately after completing the marathon. In conclusion, for the first time, it is shown that the myokines IL-8 and IL-10 are related to improvement of the performance of marathon runners presenting EIB.

## 1. Introduction

Exercise-induced bronchoconstriction (EIB) is defined as a transient narrowing of the airways that occurs after exercise in individuals with (EIB_A_) or without underlying asthma (EIB_wA_) [1,2]. Both EIBA and EIB_wA_ have peculiarities in pathogenic mechanisms, diagnostic criteria, and responses to treatment and prevention [3]. 

Regarding the pathogenic mechanisms, although at present, the cause of EIB is not entirely understood, two classical theories, i.e., the osmotic and the thermal theories, were purposed to explain its occurrence. In relation to the latter, the occurrence of vasodilation, associated with airway rewarming, plays a role in the induction of bronchial obstruction after exercise [4]. Regarding the osmotic theory, which is currently the most accepted, the hyperventilation through the mouth associated with intense exercise requires the humidification and heating of large volumes of air in a short period of time, leading to airway dryness. Water loss by evaporation in airway surfaces is associated with events that can trigger the contraction of bronchial smooth muscle, such as mast cell degranulation [5], which releases pro-inflammatory mediators that are involved in smooth muscle contractions, mucus production, and microvascular permeability, leading to airway edema and bronchoconstriction [5,6]. Furthermore, as mentioned in the review from Couto et al. [7], epithelial damage can also be involved in EIB, and several studies have demonstrated increased infiltration of eosinophils, neutrophils, and/or epithelial cells associated with EIB, as well as an increase in airway inflammatory markers. More specifically, Seys et al. [8] demonstrated an increased presence of damage-associated molecular patterns (DAMPS) in athletes with EIB. 

In terms of EIB diagnosis, it is important to clarify that the clinical history and the presence of typical symptoms (dyspnea, chest tightness, cough, and wheeze) can be used only as a complement to determine the occurrence of EIB [9], since valid diagnoses of EIB should be established though direct or indirect tests. For instance, the methacholine challenge is direct, whereas the eucapnic voluntary challenge as well as the standardized treadmill exercise test—whereby exercise may be followed by a decrease of 10 % or more in forced expiratory volume (FEV1) compared to pre-exercise levels—are indirect tests [6,7,9]. It is worthy to mention that, for some authors [10,11], the standard treadmill exercise test is preferred for EIB due to its resemblance to real-life exercise.

The diagnosis of EIB_A_ and EIB_wA_ is of great importance for people who exercise or practice sport; the disorder can manifest during/after any physical activity, and shows similar prevalence in both genders [1,2]. It is important to emphasize that this condition is frequently not recognized, often being attributed to fatigue, and, even when recognized, it does not receive the attention it deserves [12,13]. It is important to note that EIB is quite common among practitioners of endurance sports [1]. The marathon, a sport that requires great physical effort, has significantly increased in popularity in recent decades [13,14]. Several publications, including from our group, have shown a significant increase in the production of pro-inflammatory cytokines, both systemic and in the upper airways [15,16].

Based on this information, we aimed to evaluate the prevalence of EIB in a group of recreational marathon runners without asthma, as well as to investigate both systemic and upper airway inflammatory responses and their correlation with marathon performance. 

## 2. Methods 

### 2.1. Subjects and Study Design

Fifty recreational male athletes registered for the São Paulo International Marathon to be held on 17 June 2012, in the city of São Paulo, were recruited. Athletes had to meet the inclusion criteria (i.e., train at least three times per week and have completed a marathon in the last twelve months). Exclusion criteria were a history of cardiopulmonary, respiratory, or metabolic disease, the use of medication for chronic diseases, or any factor that did not permit them to run the race or finish the International Marathon of Sao Paulo.

One week later, the athletes who consented were contacted to fill in a questionnaire about smoking habits and general health conditions such as the presence/absence of cardiopulmonary or metabolic diseases. The time they usually need to complete a 10 km training run was also asked, and this was used to determine the initial treadmill speed in the cardiopulmonary test (as will be described below) and estimate the time they would need to complete the marathon. In this contact, runners had baseline measurements (see below) and were also informed about the peripheral blood sample collection, as well as the ergospirometric and spirometry tests, planned for thirty days after the marathon. From the 50 athletes recruited, two did not complete the marathon and were therefore excluded from the study; 10 athletes were excluded because they did not complete the exams, leaving the present study with a total of 38 athletes. 

All subjects gave their informed consent for inclusion before they participated in the study. The study was conducted in accordance with the Declaration of Helsinki, and the protocol was approved by the Ethics Committee of Federal University of São Paulo (0573/11).

**Figure 1:**
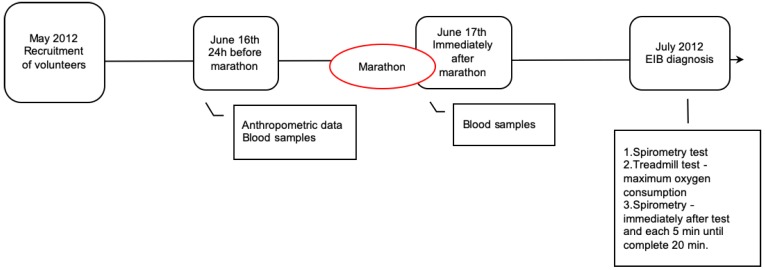
Study design.

It was an analytical cross-sectional study, with three visits on different days (Figure 1). At the first visit, all participants were submitted to baseline peripheral blood sampling and measurement of physical characteristics (weight, height, and body composition). Immediately after they completed the marathon, a second collection of peripheral blood was performed. In the last visit (30 days later), the pulmonary function was evaluated at rest and immediately after a maximal cardiopulmonary exercise test to detect EIB. The athletes were instructed to describe any respiratory symptom during the study period. All volunteers reported that they did not experience any respiratory symptoms during the study period (i.e., before, during. or after the race). The researchers were blinded for the results of the marathon during the whole period of data collection.

### 2.2. Cytokines Measurements and Cellular Counting

Plasma samples were obtained from peripheral blood collected in tubes containing ethylenediamine tetraacetic acid [(EDTA) 2 tubes of 5 mL], which were centrifuged at 900 *g* for 10 min. Plasma samples was then stored at −80 °C for later determination of circulating cytokine concentration.

Plasma IL-1β, IL-4, IL-6, IL-8, IL-10, and TNF-α concentrations were obtained by Multiplex MILLIPLEX (Merck Millipore, Burlington, MA, USA) analysis, according to manufacturer’s instructions. The concentration of high-sensitivity C-reactive protein (_HS_CRP) protein and creatine phosphokinase (CPK) were determined spectrophotometrically.

### 2.3. Evaluation of Aerobic and Pulmonary Capacity

Thirty days after the marathon, all 38 participants underwent pulmonary function testing with a computerized pneumotachograph spirometer (SpiroPro, SensorMedics, Yorba Linda, CA, USA) pre- and post- treadmill exercise test, according to the recommendations of the American Thoracic Society [17], to diagnosis of EIB. Immediately afterwards (0 min), and 5, 10, 15, and 20 min after the end of the test, the volunteers performed spirometry tests. EIB was considered positive (EIB+) when FEV1 decreased by 10% or more in relation to the baseline test in any of the spirometric tests performed after the treadmill test [17].

A maximal effort test was accomplished by all volunteers in order to determine their aerobic capacity. 

Cardiopulmonary exercise testing was performed on a treadmill coupled with a gas analyzer (FitMate ™, Cosmed, Rome, Italy) and with an electrocardiography software (TEB^®^ APEX 2000, São Paulo, Brazil). The analysis was performed using the maximal incremental treadmill protocol with a fixed slope of 1% and a 1 km/h increase every minute, until the maximum treadmill speed (18 km/h) was reached. The first minute on the treadmill was the same for all subjects, i.e., 5 km/h. In the second minute, a different speed based on the time needed to complete a 10 km run was used as described: those who reported taking less than 35 min to run 10 km started the second minute of the test at 9 km/h (group 1: 8 volunteers); those who reported needing between 36 and 45 min started the second minute of the test at 8 km/h (group 2: 8 volunteers); those who reported taking more than 46 min (maximum 1 h and 10 min) to run the 10 km started the second minute of the test at 7 km/h (group 3: 22 volunteers) [18]. When the maximum treadmill speed was reached (after 10, 11, or 12 min), a 2% inclination was added every minute (the treadmill maximum inclination capacity was 26%) until completion of test. The test was interrupted when the volunteer reported intense fatigue or exhaustion, or by the physician responsible for monitoring the tests when abnormalities on the electrocardiogram and oxygen consumption were observed. Oxygen uptake (VO_2_) was considered maximum (VO_2 max_) when a respiratory exchange ratio (RER) > 1.1 L was reached, or when the report of exhaustion was associated with reaching the maximum predicted heart rate and an RER greater than 1 L. In addition, the lactate threshold was also determined during the cardiorespiratory test based on the nonlinear increases in ventilation and changes in respiratory exchange ratio (RER) parameter.

### 2.4. Statistical Analysis

All results were assessed for testing the null hypothesis of normality using the Shapiro-Wilks test. The nonparametric baseline prerace and the postrace values from blood inflammatory markers were compared using the Wilcoxon test. The EIB negative (−) and positive (+) subjects were compared using the Mann-Whitney U test at significance level of *p* < 0.05. Spearman’s correlation was applied to analyze the correlations within each group, and the results were considered statistically significant at *p* < 0.02. Parameters such as anthropometric value, oxygen consumption, pulmonary capacity, and inflammatory marker concentrations are presented as median and percentiles.

A statistical analysis was performed using software GraphPad Prism 8 (version 8.1.2, GraphPad Software, San Diego, CA, USA).

### 2.5. Data Availability Statement

The datasets generated and/or analyzed during the current study are available from the corresponding author upon request.

## 3. Results

### 3.1. Physical Characteristics, and Pulmonary and Aerobic Capacity of All Volunteers

Table 1 presents a description of the anthropometric data, aerobic capacity, and pulmonary function of all volunteers.

Concerning the cardiopulmonary exercise test to evaluate the maximal oxygen uptake, it is worth mentioning that only five volunteers did not reach the endpoint criterion of RER ≥ 1.1 L (gold standard) for a maximal test and discontinued the test due to muscle fatigue, despite the fact that they had reached the predicted maximal heart rate and a RER that was greater than 1 L. Bearing in mind that these five volunteers reached the secondary endpoint criteria for maximal oxygen uptake, they remained in the study. 

### 3.2. Exercise-Induced Bronchoconstriction Diagnosis, Pulmonary and Aerobic Capacity, and Performance of Volunteers Presenting or not EIB

In order to determine the presence or absence of EIB, a reduction of at least 10% in FEV1 after the treadmill cardiopulmonary test [17] was used; it was found that 9 (23.6%) volunteers presented EIB (EIB+), according to data shown in Table 2 and Supplementary Material (Table S1). In the EIB− group, 3 subjects presented borderline values (decreases of 9%, 8%, and 7%) in maximal fall in FEV1 after treadmill test (Table S1). During the test, no volunteers reported respiratory distress or any symptom requiring medical or drug intervention during or after the treadmill test. None of the 5 volunteers who did not reach a RER ≥ 1.1 L was diagnosed with EIB. In addition, no differences were found in the anthropometric data when the volunteers were separated into EIB+ and EIB− groups.

**Figure 2:**
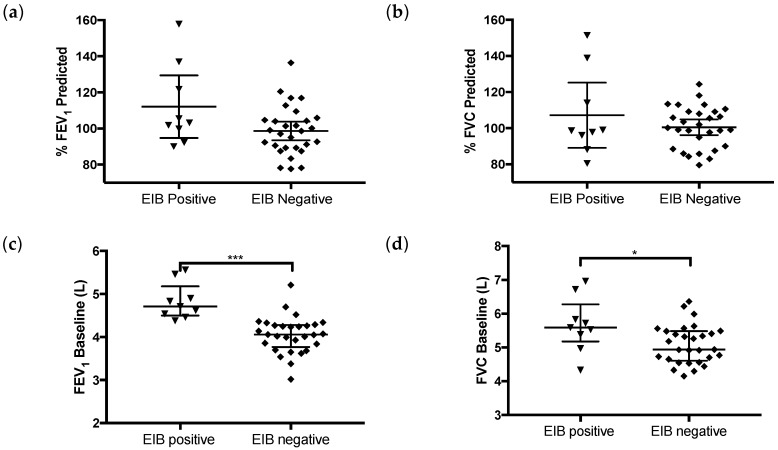
Comparison between groups of baseline values of: (**a**) % predicted FEV1 (*p* = 0.09); (**b**) % predicted FVC (*p* = 0.93); (**c**) FEV1 (*p* ≤ 0.001); (**d**) FVC (*p* = 0.02). * < 0.05 and *** < 0.001.

Both the % predicted FEV1 (median EIB+ 103.2 and EIB− 98.6; *p* = 0.09) and FVC (median EIB+ 98.6 and EIB− 100.2; *p* = 0.93) were similar between both groups. Indeed, the comparison between the percentage of the predicted values of FEV1 and FVC reached in the baseline test did not present any difference. By contrast, as presented in Figure 2, the absolute values of FEV1 and FVC between EIB+ and EIB− subjects were significantly different at baseline, with higher values in EIB+ subjects. 

**Figure 3:**
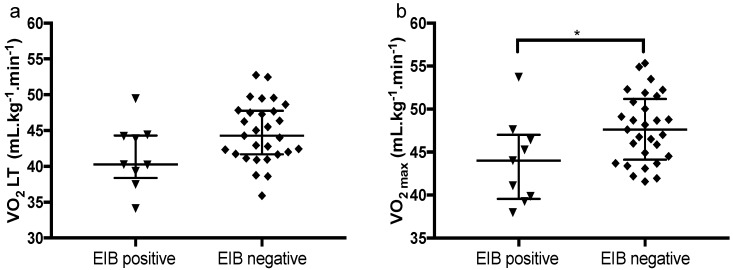
Comparison of aerobic capacity between groups. (**a**) oxygen consumption in lactate threshold (*p* = 0.06). (**b**) maximal oxygen consumption (*p* = 0.03). * < 0.05.

VO_2 max_, on the other hand, was significantly lower in EIB+ subjects (Figure 3), whereas the values obtained in the lactate threshold were similar.

In spite of the difference found in VO_2max_ between groups, the marathon finishing time was similar between both groups (EIB+ 272.6 min ± 43.47; EIB− 266.4 min ± 47.57; *p* = 0.69). 

**Figure 4:**
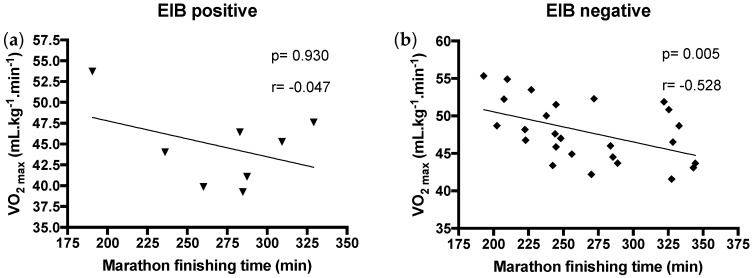
Correlation between aerobic capacity and performance. (**a**): EIB positive; (**b**): EIB negative.

We next correlated the marathon finishing time with the VO_2max_. The analysis of performance and aerobic capacity, as expected, showed a moderately negative correlation between maximal oxygen uptake and marathon finishing time for EIB– subjects (Figure 4b). However, this was not the case for EIB+ subjects (Figure 4a). 

The correlation result for oxygen uptake in lactate threshold (VO_2_ LT) and time to finish the marathon showed a similar result, with a negative correlation between both in EIB– but not in EIB+ subjects (EIB+ *p* = 0.7, r = −0.166 and EIB− *p* = 0.03, r = −0.414). To rule out that the absence of a negative correlation in EIB+ subjects was due to the small number of volunteers in this group, the correlation analysis was also performed placing all subjects within a single group. Although a significant negative correlation between VO_2max_ and time to finish a marathon could still been found (*p* = 0.003), the correlation strength decreased (r = −0.481 compared to r = −0.531). Similar data were seen when VO_2_ LT was compared with the same correlation in the complete group (*p* = 0.03, r = −0.414 compared to *p* = 0.03; r = −0.365). This argues against the fact that the correlation in EIB+ subjects was not observed due to the low subject number.

### 3.3. Inflammatory Markers

Cytokine levels, and CPK and _HS_CRP concentrations in blood were compared between both groups. Table 3 presents the comparisons between the levels of blood inflammatory markers before (24 h before, i.e., baseline) and immediately after the marathon for each group. IL-6, IL-8, IL-10, TNF-α, and CPK levels were significantly increased after the race, compared to baseline levels in both groups. However, no differences were found for IL-1β or _HS_CRP levels between baseline and after running in any group. IL-4 values were below the detection level in several samples, and as a result, no comparison between baseline levels and levels after running could be made.

When comparing baseline data between both groups, no differences could be observed in any parameter. Similarly, data immediately after running did not differ between both groups (Table 3).

The correlations between marathon finishing time and specific blood inflammatory markers, in order to investigate whether or not the presence of EIB changes specific correlations, are shown in Table 4.

In the EIB+ subjects, a strong negative correlation was observed between the time to finish the marathon and the chemokine IL-8 and cytokine IL-10 levels immediately after the race. A trend towards a positive correlation (*p* = 0.058) was observed between marathon finishing time and level of sensitive C-reactive protein.

In the EIB– group, no statistically significant correlation between time to finish the marathon and inflammatory markers was found, either baseline or after the race, but a tendency (*p* = 0.061) towards a positive correlation between time to finish the marathon and the concentration of IL-1β immediately after the race was observed in the EIB− group.

## 4. Discussion

The present study showed that the prevalence of EIB among enrolled marathon runners was 23.6%, which is in agreement with other studies [19,20,21,22]. Based on this finding, the volunteers were distributed into two groups (EIB+ and EIB−), allowing us to evaluate the differences in pulmonary and aerobic capacities, as well as in the inflammatory response between these groups, in order to correlate such responses with marathon performance. Higher FVC and FEV1 absolute values were found in the EIB+ group compared to the EIB– group at baseline, even though the percentage of predicted values did not show significant differences. In relation to the aerobic capacity, the EIB+ group showed lower maximal oxygen uptake compared to EIB– group, whereas the aerobic threshold and time to finish the marathon were similar between the groups. As expected, the EIB– group showed a negative correlation between maximal oxygen uptake and its threshold; however, this result was not observed in the EIB+ group. Regarding the inflammatory response, an increase of systemic levels of IL-6, IL-8, IL-10, TNF-α, and CPK was observed immediately after the marathon ending in both groups, compared to the baseline values. In addition, negative correlations were also observed between circulatory levels of IL-8 and IL-10 and marathon ending immediately after the marathon only in the EIB+ group.

The novelty of this study was that the marathon performance found in the EIB+ group was not determined by the maximal aerobic capacity or its threshold, but could be influenced by the systemic cytokine levels, highlighting the production of IL-8 and IL-10 during the marathon.

It is worth highlighting that none of the EIB+ volunteers reported any typical subjective symptom of EIB during or prior to the marathon or during the exercise test, which is typical for mild EIB (reduced FEV1 among 10–15%). This observation can be explained by the fact that, clinically, the symptoms of EIB might be confused with typical symptoms of exhaustion/dyspnea during exercise as a result of performing exercise at such intensive level, such as in a marathon. Nevertheless, the percentage of EIB+ found here in relation to marathon runners is in agreement with other studies [22,23], and is higher than in the general population [19,20,21,23]. 

Interestingly, the marathon finishing times found in both groups were similar. In this regard, the higher pulmonary capacity at baseline observed in the EIB+ group compared to the EIB– group helped them to obtain a similar aerobic threshold as that found in the EIB– group, even after a fall in their pulmonary capacity and a diminished maximal oxygen uptake. According to the literature, maximal oxygen uptake and its threshold are the most common parameters evaluated in exercise physiology, and are used as a basis for the prediction of aerobic performance and for training prescriptions for athletes [24,25]. Our study corroborates the well-known concept that VO_2 max_ and its threshold predicts athletes’ performance (e.g., marathon finishing time) [24,25,26,27]. However, this prediction pattern was found only in the EIB– group. Although this observation cannot be directly explained by the physiological aspects evaluated in this study, the differences in the correlation between the cytokines and marathon finishing time provide some level of understanding of this phenomenon, or at least, open a new route of investigation.

Cytokines are proteins that present many functions, such as the regulation of inflammatory responses, chemotaxis, and cell activation, among others [28,29]. During physical exercise, muscle contractions elicit the release of several molecules, including cytokines such as IL-1ra, IL-6, IL-8, IL-10, and TNF-α [30,31]. In this situation the cytokines released from the muscle in contraction are known as myokines [30]. Therefore, the higher levels of IL-6, IL-8, IL-10, and TNF-α found in this study were expected after a marathon [31,32]. Concerning IL-1β, although some studies have shown increases in its circulating level after a marathon, in other studies, its level remained unchanged [31,33,34,35]. Even though these results were found in both volunteer groups, significant differences were observed when the cytokines levels, specifically IL-8 and IL-10, were correlated with marathon finishing time. 

IL-8 is a classical proinflammatory cytokine that belongs to a subfamily of CXC chemokines. It was originally identified as a chemoattractant factor for neutrophils [36,37]. Beyond its proinflammatory role, IL-8 also presents a prominent angiogenic function and, as mentioned, is considered a myokine [38,39]. According to the literature [39], the increased IL-8 levels in response to muscle damage induced by a physical exercise session are mainly associated with the regulation of muscle angiogenesis by its binding to the CXC receptor 2 (CXCR2) expressed in microvascular endothelial cells in order to improve muscle regeneration [40]. In addition, it has been suggested that the release of greater amounts of IL-8 from muscle can occur concomitant with increases in the circulatory CPK levels postexercise, as found here, in order to mitigate muscle soreness [39]. Based on these data, we suggest that the higher IL-8 levels observed in both groups can putatively ameliorate the oxygen and substrate delivery to the muscle during the marathon. In the EIB+ group, a significant negative correlation between IL-8 levels and marathon finishing time supports our suggestion, since that volunteers in this group with better performance showed higher levels of this myokine. It is worth mentioning that, at this time, no studies have analyzed the effect of IL-8 in the context evaluated in the present study. Although Chimenti et al. [41] showed that the occurrence of acute bronchial epithelial damage after a half-marathon was associated with higher IL-8 levels, it is important to clarify that the increased levels of this cytokine were found in sputum supernatants, and not systemically. Furthermore, in a general way, according to the literature, the presence of IL-8 in airways is mainly associated with impaired pulmonary function. However, the overwhelming majority of these studies showed that an association between IL-8 and impaired pulmonary function occurs in chronic lung diseases [42,43,44]. Therefore, although we were not able to assess the levels of IL-8 in the airways of the individuals participating in this study, the observation that both groups had similar marathon finishing times allows us to suggest that, even if IL-8 induced any impairment of lung function, it was not enough to affect the performance in the EIB+ group.

Another factor that may influence the similar performance observed between the two groups is the higher circulant IL-10 levels found immediately after the marathon. It is well-known that IL-10 has a potent anti-inflammatory and proresolving properties [45] and, in the airways, can act as a protective factor against mucosal inflammation [46]. 

In relation to physical exercise, it has been reported that the IL-6 released by strenuous exercise precedes the release of IL-10, in order to regulate the systemic inflammation induced by the exercise [33]. Chen et al. [47] reported remarkable increases in the secretions of myokines, such as IL-8 and IL-10 derived from contraction of cultured myotubes, characterizing IL-10 as a myokine [47,48]. At basal condition, the secretion of myokines, such as IL-6 and IL-8, was also observed in a circadian manner; however, this was not the case with IL-10 levels [49]. Therefore, the observation of higher circulating IL-6 levels in both groups may underly the observed elevation of IL-10 levels. In relation to marathons, our group previously demonstrated that marathon runners who had increased IL-10 levels in the circulation immediately after the marathon and also in the nasal mucosa 72 hours after the marathon did not present upper respiratory symptoms [12,33]. In addition, it has been also reported that systemic IL-10 can modulate the pulmonary function by modulating the inflammatory response of the airways [29,45], including that associated with physical exercise [50,51]. However, studies that aimed to evaluate the effect of IL-10 in the context of EIB are scarce [52,53]. So, for the first time, we observed that circulating IL-10 levels were negatively correlated with marathon finishing times in the EIB+ group. Taking in account the properties of IL-10, we suggest that the marathon runners in the EIB+ group with higher circulating IL-10 levels were able to finish the race earlier than those with lower IL-10 levels probably due to better modulation of systemic inflammation, and also by diminishing the negative impact of the reduction in pulmonary function imposed by the exercise and the reduced aerobic capacity, as compared with the EIB– group.

Regarding the limitations of the study, a point to be considered is the small number of volunteers, and also the differences between the number of volunteers in each group (EIB+ and EIB–). It is important to mention that most studies that have aimed to evaluate EIB, both in the general population and in practitioners of sports, presented results that included asthmatics; this was not the case in our study, since one of the objectives of this study was to assess the prevalence of EIB in a group of recreational marathoners without asthma. Therefore, although it is known that the number of participants enrolled in a study can impact the power of the statistical tests, the number of volunteers participating in this study was sufficient to achieve the relevant statistical differences to mitigate this study limitation. Another limitation of this study is associated to the absence of assessments concerning to the pulmonary inflammatory condition of the volunteers both before and immediately after the race. In this way, it has been demonstrated that the evaluation of fractional exhaled nitric oxide (FeNO), a remarkable biomarker presenting increased levels in association with pulmonary inflammation, can be useful for monitoring pulmonary inflammatory status in different situations [54,55,56], including in the context of running [46,57]. Other points that deserve to be mentioned are related to the inclusion of only men in this study, the age of the participants, and also, to the environmental conditions present during the marathon event [58]. With regard to the inclusion of only men, although the literature shows that the participation of women in marathons has increased, leading to a decrease in the proportion of men to women, the number of men participating in these races is still greater than that of women. In addition, we decided to evaluate only men in order to avoid significant variation of sex hormones, which, especially in women, can influence physical exercise practice and performance, as reported by Foster et al. [59]. Finally, it is also important to note that, in agreement with the literature, the environmental conditions during the race can also influence inflammatory responses [60,61]; the opportunity to evaluate numerous volunteers who participated in the same marathon minimizes this issue.

## 5. Conclusions

In conclusion, the results of this study demonstrate that the percentage of marathon runners presenting EIB was in agreement to the current literature, and that the increased levels of myokine IL-10, by its capacity to reduce the systemic inflammation induced by the strenuous and prolonged exercise, such as a marathon, may influence the maintenance of pulmonary function. Taken together, our results showed that a relationship between myokines and performance was presented in EIB+ marathon runners, and that this could lead them to achieve similar levels of performance as EIB– marathon runners. Further studies are needed to clarify the role of IL-8 and IL-10 myokines in the performance, pulmonary function, and muscle metabolism of EIB+ marathon runners.

## Figures and Tables

**Table 1:** Anthropometric data, aerobic capacity, and pulmonary function of all volunteers—International Marathon of Sao Paulo 2012.

Parameter	Minimum	25% Percentile	Median	75% Percentile	Maximum
Age (years)	24.0	33.0	38.5	44.0	56.0
Height (cm)	166.5	172.0	175.5	180.3	193.5
Weight (kg)	56.2	69.3	74.7	81.6	101.4
Fat (%)	3.0	15.3	18.6	20.8	25.7
Body mass index	19.0	22.4	24.6	25.9	30.3
Predicted max HR (beats/min)	164.0	176.0	181.5	187.0	196.0
Maximal heart rate (beats/min)	167.0	180.0	185.5	191.0	206.0
% of predicted max HR (%)	91.0	99.0	102.0	16.3	113.0
Duration of test (min)	7.7	10.1	11.5	12.0	13.8
VO_2 max_ (mL kg^−1^ min^−1^)	38.0	43.6	46.6	50.2	55.3
VO_2_ aerobic threshold (mL kg^−1^ min^−1^)	28.7	32.7	34.9	38.4	47.6
VO_2_ lactate threshold (mL kg^−1^ min^−1^)	34.1	40.9	44.0	47.6	52.7
Maximal ventilation (L)	111.6	139.6	153.2	170.2	193.5
Maximal respiratory exchange ratio (L)	1.0	1.1	1.2	1.2	1.4
Marathon finishing time (min)	190.7	233.8	265.1	312.5	344.6
Predicted FVC (L)	4.4	4.8	5.1	5.5	6.3
FVC baseline (L)	4.2	4.7	5.3	5.6	7.0
% of predicted FVC baseline (%)	79.6	89.7	99.1	109.6	151.4
FVC 0 min (L)	4.1	4.6	5.0	5.4	6.3
FVC 5 min (L)	4.0	4.7	5.1	5.5	6.6
FVC 10 min (L)	3.7	4.7	5.1	5.4	6.4
FVC 15 min (L)	3.5	4.7	5.2	5.4	6.8
FVC 20 min (L)	4.2	4.7	5.2	5.5	6.4
Predicted FEV1 (L)	3.4	3.8	4.2	4.5	5.2
FEV1 baseline (L)	3.0	3.9	4.2	4.5	5.6
% of predicted FEV1 baseline (%)	77.7	90.5	100	106.8	157.8
FEV1 0 min (L)	3.2	3.9	4.2	4.5	5.8
FEV1 5 min (L)	3.3	3.9	4.1	4.4	5.7
FEV1 10 min (L)	3.2	3.8	4.2	4.4	5.3
FEV1 15 min (L)	3.3	3.8	4.1	4.4	5.6
FEV1 20 min (L)	3.2	3.8	4.2	4.5	5.4

Note: FVC (Forced vital capacity), FEV1 (Forced expiratory volume in one second)

**Table 2:** FEV_1_ absolute and relative values of all EIB positive volunteers—International Marathon of Sao Paulo 2012.

Volunteer	Absolut Values (L)						Relative Values (%)					Difference
ID #	FEV1 Baseline	FEV1 0 min	FEV1 5 min	FEV1 10 min	FEV1 15 min	FEV1 20 min	FEV1 0 min	FEV1 5 min	FEV1 10 min	FEV1 15 min	FEV1 20 min	FEV1 % Maximal Fall
7	5.46	3.72	4.04	3.57	3.65	3.57	68	74	65	67	65	−35
12	4.83	4.24	3.72	4.02	3.74	4.6	88	77	83	77	95	−23
6	4.38	3.95	3.87	3.45	3.66	4.48	90	88	79	84	102	−21
25	5.56	4.63	4.43	4.73	5.57	5.39	83	80	85	100	97	−20
32	4.54	3.75	3.66	3.73	3.75	3.75	83	81	82	83	83	−19
24	4.46	4.21	4.79	4.1	4.08	3.73	94	107	92	91	84	−16
9	4.62	4.31	4.05	4.01	3.94	3.92	93	88	87	85	85	−15
8	4.9	4.53	4.59	4.23	4.75	4.41	92	94	86	97	90	−14
49	4.71	4.52	4.2	4.33	4.43	4.55	96	89	92	94	97	−11

Note: The highlighted values indicate the moments when the drop in FEV1 was equal to or higher than 10%.

**Table 3:** Comparison of blood inflammatory markers before and after the race and between groups—International Marathon of 2012.

Variables	EIB Positive (*n* = 9)			EIB Positive (*n* = 29)		
	Baseline	Immediately after		Baseline	Immediately after	
**IL-1β (pg/mL)**	0.02 [0.01–0.21]	0.13 [0.00–0.27]		0.14 [0.05–0.27]	0.20 [0.13–0.42]	
**IL-4 (pg/mL)**	0.00 [0.00–0.00]	0.00 [0.00–2.30]		0.00 [0.00–0.80]	0.00 [0.00–4.68]	
**IL-6 (pg/mL)**	0.03 [0.00–0.12]	23.23 [20.48–32.16]	*	0.08 [0.03–0.18]	28.81 [18.41–52.38]	*
**IL-8 (pg/mL)**	1.62 [1.37–3.38]	15.28 [7.35–19.83]	*	2.74 [1.70–4.89]	16.53 [10.35–24.46]	*
**IL-10 (pg/mL)**	0.06 [0.00–1.14]	64.13 [15.88–262.5]	*	0.27 [0.07–0.93]	80.83 [30.80–215.90]	*
**TNF-α (pg/mL)**	5.78 [4.52–7.95]	12.32 [9.39–15.19]	*	7.95 [5.99–9.43]	11.07 [9.04–15.52]	*
**CPK (U/L)**	145.0 [93.5–454.0]	496.0 [197.5–979.5]	*	309.0 [134.0–261.8]	490.5 [328.3–710.0]	*
**_HS_PCR (mg/dL)**	0.12 [0.07–0.19]	0.15 [0.08–0.25]		0.07 [0.02–0.13]	0.09 [0.03–0.15]	

Note: The IL-4 cytokine concentration values for most of the samples were undetectable, which made it impossible to carry out a statistical analysis. No differences were found in the comparison between EIB+ and EIB– groups’ values for baseline or immediately after the marathon. * Statistically significant difference in comparison to baseline value.

**Table 4:** Correlations between marathon finishing time and blood markers of all volunteers and by groups. International Marathon of Sao Paulo 2012.

Parameters	EIB Positive		EIB Negative	
	Marathon Finishing Time		Marathon Finishing Time	
	r	*p*	r	*p*
IL-1β Baseline	0.4671	0.246	0.0271	0.898
IL-1β Immediately after	0.6138	0.115	0.3807	0.061
IL-4 Baseline	−0.2474	0.750	0.0316	0.881
IL-4 Immediately after	−0.1559	0.750	0.2054	0.325
IL-6 Baseline	0.5279	0.186	0.4387	0.028
IL-6 Immediately after	−0.4286	0.299	0.1436	0.494
IL-8 Baseline	0.0476	0.935	0.1267	0.546
IL-8 Immediately after	−0.8095	0.022	−0.0535	0.800
IL-10 Baseline	0.0488	0.928	0.0768	0.715
IL-10 Immediately after	−0.9762	≤0.001	−0.2718	0.189
TNF-α Baseline	0.5952	0.132	−0.0359	0.865
TNF-α Immediately after	−0.3095	0.462	−0.1471	0.483
CPK Baseline	0.5476	0.171	−0.0551	0.794
CPK Immediately after	0.6190	0.115	0.1121	0.594
_HS_CRP Baseline	0.6071	0.167	0.0027	0.990
_HS_CRP Immediately after	0.7143	0.058	0.0222	0.916

Note: significance level ≤0.02.

## Supplementary Materials

The following are available online at https://www.mdpi.com/1660-4601/17/8/2622/s1, Table S1: FEV1 absolute and relative values of all EIB negative volunteers—International Marathon of Sao Paulo 2012.
