# Supplementary material for: The Relationship of IL-8 and IL-10 Myokines and Performance in Male Marathon Runners Presenting Exercise-Induced Bronchoconstriction
Source: Int J Environ Res Public Health. 2020 Apr 11;17(8):2622. doi: 10.3390/ijerph17082622 (PMC7215610; doi:10.3390/ijerph17082622)
Supplement: Supplementary file 1 [file ijerph-17-02622-s001.pdf]

**Table S1.** $FEV_1$ absolute and relative values of all EIB negative volunteers - International Marathon of Sao Paulo 2012.

| | Absolut values (L) | | | | | | Relative values (%) | | | | | |
|---|---|---|---|---|---|---|---|---|---|---|---|---|
| ID # | $FEV_1$ Baseline min | $FEV_1$ 0min | $FEV_1$ 5min | $FEV_1$ 10min | $FEV_1$ 15min | $FEV_1$ 20min | $FEV_1$ 0min | $FEV_1$ 5min | $FEV_1$ 10min | $FEV_1$ 15min | $FEV_1$ 20min | $FEV_1$ % Maximal fall |
| 31 | 3.62 | 3.73 | 3.62 | 3.3 | 3.78 | 3.65 | 103 | 100 | 91 | 104 | 101 | -9 |
| 19 | 4.05 | 4.92 | 3.99 | 3.72 | 3.85 | 3.81 | 121 | 99 | 92 | 95 | 94 | -8 |
| 28 | 3.68 | 3.63 | 3.43 | 3.62 | 3.53 | 3.66 | 99 | 93 | 98 | 96 | 99 | -7 |
| 42 | 4.36 | 4.29 | 4.15 | 4.34 | 4.08 | 4.6 | 98 | 95 | 100 | 94 | 106 | -6 |
| 38 | 5.21 | 5.79 | 5.22 | 5.12 | 5.19 | 4.9 | 111 | 100 | 98 | 100 | 94 | -6 |
| 2 | 4.24 | 4.16 | 3.99 | 5.27 | 4.3 | 4.21 | 98 | 94 | 124 | 101 | 99 | -6 |
| 20 | 3.54 | 3.55 | 3.55 | 3.57 | 3.52 | 3.41 | 100 | 100 | 101 | 99 | 96 | -4 |
| 13 | 4.14 | 4.21 | 3.99 | 4.31 | 4.19 | 4.3 | 102 | 96 | 104 | 101 | 104 | -4 |
| 17 | 4.06 | 4.56 | 4.02 | 3.96 | 3.95 | 3.93 | 112 | 99 | 98 | 97 | 97 | -3 |
| 30 | 4.25 | 4.2 | 4.19 | 4.97 | 4.73 | 4.25 | 99 | 99 | 117 | 111 | 100 | -1 |
| 40 | 4.29 | 4.23 | 4.24 | 4.3 | 4.41 | 4.48 | 99 | 99 | 100 | 103 | 104 | -1 |
| 14 | 3.65 | 3.71 | 3.6 | 3.65 | 3.62 | 3.65 | 102 | 99 | 100 | 99 | 100 | -1 |
| 46 | 4.33 | 4.49 | 4.35 | 4.28 | 4.35 | 4.32 | 104 | 100 | 99 | 100 | 100 | -1 |
| 43 | 4.27 | 4.23 | 4.24 | 4.31 | 4.38 | 4.38 | 99 | 99 | 101 | 103 | 103 | -1 |
| 22 | 4.01 | 3.98 | 4.04 | 4.08 | 4.05 | 4.05 | 99 | 101 | 102 | 101 | 101 | -1 |
| 34 | 4.52 | 4.49 | 4.58 | 4.71 | 4.56 | 4.63 | 99 | 101 | 104 | 101 | 102 | -1 |
| 45 | 4.7 | 4.91 | 4.77 | 4.75 | 4.67 | 4.79 | 104 | 101 | 101 | 99 | 102 | -1 |
| 18 | 3.86 | 3.91 | 3.92 | 3.86 | 3.86 | 3.93 | 101 | 102 | 100 | 100 | 102 | 0 |
| 48 | 3.99 | 4.34 | 4.01 | 4.02 | 4.05 | 4.17 | 109 | 101 | 101 | 102 | 105 | 1 |
| 37 | 4.34 | 4.4 | 4.38 | 4.49 | 4.44 | 4.37 | 101 | 101 | 103 | 102 | 101 | 1 |
| 26 | 3.84 | 3.88 | 3.88 | 3.99 | 3.96 | 3.99 | 101 | 101 | 104 | 103 | 104 | 1 |
| 15 | 4.23 | 4.34 | 4.3 | 4.31 | 4.35 | 4.28 | 103 | 102 | 102 | 103 | 101 | 1 |
| 23 | 3.93 | 3.99 | 4.25 | 4.05 | 4.11 | 4.04 | 102 | 108 | 103 | 105 | 103 | 2 |
| 3 | 4.02 | 4.1 | 5.69 | 4.76 | 4.46 | 4.14 | 102 | 142 | 118 | 111 | 103 | 2 |
| 1 | 3.7 | 3.98 | 3.93 | 3.85 | 3.89 | 3.99 | 108 | 106 | 104 | 105 | 108 | 4 |
| 33 | 4.07 | 4.26 | 4.34 | 4.28 | 4.25 | 4.69 | 105 | 107 | 105 | 104 | 115 | 4 |
| 16 | 4.26 | 4.63 | 4.47 | 4.48 | 4.53 | 4.54 | 109 | 105 | 105 | 106 | 107 | 5 |
| 10 | 3.02 | 3.21 | 3.28 | 3.24 | 3.26 | 3.2 | 106 | 109 | 107 | 108 | 106 | 6 |
| 21 | 3.38 | 3.96 | 4.19 | 4.27 | 3.79 | 3.64 | 117 | 124 | 126 | 112 | 108 | 8 |
